# Development and evaluation of a dynamic nomogram model for intraoperative blood transfusion decision-making

**DOI:** 10.3389/fmed.2025.1566325

**Published:** 2025-06-13

**Authors:** Min Li, Wei Jiang, Jialing Lin, Hui Du, Jiawen Shan, Li Qin

**Affiliations:** ^1^Department of Blood Transfusion Medicine, Sichuan Tianfu New Area People’s Hospital, Chengdu, China; ^2^Department of Clinical Laboratory Medicine, Sichuan Tianfu New Area People’s Hospital, Chengdu, China; ^3^Department of Blood Transfusion Medicine, West China Hospital, Chengdu, China

**Keywords:** blood transfusion decision-making, nomogram modeling, Lasso regression, peroperative, transfusion

## Abstract

**Objective:**

By gathering data on patients with intraoperative blood transfusion and investigating the factors influencing intraoperative blood transfusion in patients, we aimed to construct a dynamic nomogram decision-making model capable of continuously predicting the probability of intraoperative blood transfusion in patients. This was done to explore a new mode of individualized and precise blood transfusion and to guide doctors to make timely and reasonable blood transfusion decisions and save blood resources.

**Methods:**

Data of surgical patients in our hospital from 2019 to 2023 were collected. Among them, 705 patients who had blood transfusions were the experimental group, and 705 patients without intraoperative blood transfusions were randomly selected as the control group. Preoperative and intraoperative indicators of 1,410 patients were collected. 80% of the data set was used as the training set and 20% as the test set. In the training set, independent risk factors affecting intraoperative blood transfusion in patients were obtained through Lasso regression and binary logistic regression analysis, and the regression model was established and validated.

**Results:**

Through Lasso regression with cross-validation and binary logistic regression analysis, the independent risk factors affecting patients’ intraoperative blood transfusion decision-making were determined as ASAs (III) (OR = 3.009, 95% CI = 1.311–6.909), surgical grading (IV) (OR = 3.772, 95% CI = 1.112–12.789), EBL (OR = 1.003, 95% CI = 1.002–1.004), preHGB (OR = 0.932, 95% CI = 0.919–0.946), LVEF (OR = 1.063, 95% CI = 1.028–1.098), Temp (OR = 57.14, 95% CI = 9.740–35.204), preAPTT (OR = 1.147, 95% CI = 1.079–1.220), and preDD (OR = 1.127, 95% CI = 1.048–1.212). The area under the curve (AUC) of the receiver operating characteristic curve (ROC) of the training set was 0.983, *p* < 0.05. By calculating the Jordon index, when the Jordon index reached its maximum value, the corresponding diagnostic probability threshold was 0.515. When the model predicted that the probability threshold was 0.515, the sensitivity was 0.939 and the specificity was 0.964. These independent risk factors were introduced into R statistical software to fit the intraoperative blood transfusion decision-making dynamic nomogram model. The goodness of fit test of the model for the training set was *χ*^2^ = 111.85, *p* < 0.01, and the AUCs of the training set and the testing set were 0.983 and 0.995, respectively, *p* < 0.05. The calibration curve showed that the predicted probability of the model was in good agreement with the observed probability. Clinical decision curves (CDA) and clinical impact curves were plotted to evaluate the clinical utility of the model and the net benefit of the model.

**Conclusion:**

Variables were screened by Lasso regression, the model was developed by binary logistic regression, and a dynamic nomogram model for intraoperative transfusion decision-making was successfully fitted using R software. This model had good goodness of fit, discrimination, and calibration. The model can dynamically and instantaneously predict the probability of blood transfusion and its 95% confidence interval under the current patient indicators by selecting the patient’s independent risk factors in the drop-down mode during the operation. It can assist doctors in making a reasonable blood transfusion decision quickly and save blood resources.

## 1 Introduction

As an important therapeutic measure, blood transfusion provides protection for patients’ life safety and is widely used in clinical practice. At the same time, blood transfusion is also a double-edged sword, and the benefits of blood transfusion therapy can only be maximized by choosing the right blood products at the right time and transfusing them to the right patients in the right dosages (1). Inappropriate transfusion therapy may pose a greater risk to patients, and there is a large body of literature reporting that allogeneic blood transfusion may cause fever, allergies, and many other risks to patients (2, 3). Patients with hemoglobin between 70 and 100 g/L are determined according to the patient’s degree of anemia, cardiopulmonary compensatory function, the presence of an increased metabolic rate, and age. How exactly do clinicians make decisions about blood transfusions based on the above risks? At present, there are no specific quantitative norms in the clinic; whether the need for blood transfusion and the amount of blood transfusion can only be based on the experience of clinicians to make decisions, clinicians have a looser grasp of the transfusion trigger, resulting in the irrational use of blood.

In recent years, many scholars have devoted themselves to the research of perioperative blood management, and many research results have been achieved. Professor Liu Jin’s team at West China Hospital of Sichuan University took the lead in establishing the Perioperative Transfusion Trigger Score (POTTS), which is based on the rate of epinephrine infusion, oxygen concentration, basal metabolic rate, and angina to calculate the minimum hemoglobin concentration required to maintain a balance between the patient’s supply and oxygen consumption. A number of studies have shown that the application of the POTTS can effectively reduce the intraoperative transfusion rate and blood transfusion volume, as well as reduce postoperative complications and postoperative mortality and reduce the hospitalization costs of patients (4–7). While the application of the scale is based on the patient’s hemoglobin test value, which reflects the patient’s hemoglobin concentration at the time of blood draw, the application of the POTTS scale to patients with acute, sustained blood loss is dependent on continuous bedside hemoglobin testing, which may be difficult to achieve in primary care settings, limiting the application of the scale.

In recent years, restrictive transfusion strategy has been gradually applied to clinical practice, and the decision point for transfusion in restrictive transfusion strategy is hemoglobin 70–80 g/L. More studies have shown that a restrictive transfusion strategy can reduce perioperative transfusion, reduce the risk of rebleeding, and not increase the risk of a poor prognosis compared with an open transfusion strategy (8–11), and it is also a more effective and economical blood transfusion strategy. However, there is no specific standard for the transfusion trigger of this strategy, and most researchers have set it at 70–80 g/L. This strategy also does not consider the patient’s cardiopulmonary function, basal metabolic rate, or other factors that affect the balance of blood oxygen supply and fails to achieve an individualized blood transfusion. Some studies have shown a higher risk of using restrictive transfusion strategies for patients with American Society of Anesthesiologists scores of grade III or higher (12).

In this study, we added preoperative as well as intraoperative metrics that may affect a patient’s intraoperative blood transfusion to the restrictive transfusion strategy in an attempt to create an individualized, continuously monitored, dynamic columnar chart model that will help physicians make rapid intraoperative transfusion decisions and conserve blood resources.

## 2 Data and methods

### 2.1 Variable collection

The transfusion information management system was used to collect the preoperative variables of patients undergoing elective surgery. Preoperative variables: age, gender, body weight (W), preoperative hemoglobin concentration (preHGB), left ventricular ejection fraction (LVEF), lung function indexes (PCO_2_, SaO_2_), liver function indexes (TP, ALT, AST, and TBIL), coagulation index (prePLT, prePT, preAPTT, preINR, preTT, preFib, and preDD), kidney function (Cr, eGFR), blood type, and other indexes. Intraoperative variables: intraoperative temperature (Temp), estimated intraoperative blood loss (EBL), crystalloid input, shock index, heart rate, blood pressure, surgical grade, anesthesia grade (ASA), and so on. Temp was recorded at the start of transfusion for transfused patients and at the start of surgery for non-transfused patients. All data were organized using Excel.

### 2.2 Study subjects

The study subjects are patients undergoing elective surgery in our hospital from 2019 to 2022. A total of 1,410 cases. The inclusion criteria were (1) elective surgery patients aged 18–75 years; (2) preoperative Hb greater than 60 g/L; and (3) patients’ permanent residence at an altitude of less than 2,500 meters and complete pre- and post-surgical data. Exclusion criteria: (1) cardiac surgery, burn surgery, intraoperative uncontrollable major blood loss; (2) patients with severe blood disorders (hemophilia, hemolytic anemia, thalassemia, iron-deficiency anemia, megaloblastic anemia, and aplastic anemia); and (3) American Society of Anesthesiologists scores (grades V–VI). After 1,410 patients passed the inclusion/exclusion criteria, a total of 1,017 patients were included in the study, including 422 transfused patients and 595 non-transfused patients.

### 2.3 Statistical methods

All data were collated using Microsoft Excel and processed using SPSS 25 statistical software. Count data were expressed as the number of cases or percentage and analyzed by the *χ*^2^ test; measurement data were expressed as the mean standard deviation and analyzed by *t*-test or non-parametric test. Forest plots and ROC curves were drawn using GraphPad Prisms, and RStudio statistical software was used to complete the fitting of the dynamic nomogram model, the Hosmer goodness-of-fit test, and to draw calibration correction curves, clinical decision curves (CDA), and clinical impact curves. All analyses were performed with *α* = 0.05 as the test level and *p* < 0.05 as the difference was statistically significant.

## 3 Research results

### 3.1 Baseline patient characteristics

Baseline characteristics of 1,017 patients with 422 (41.5%) intraoperative erythrocyte transfusions and 595 (58.5%) intraoperative non-transfused erythrocytes are shown in Table 1. All variables did not conform to normal distribution by the normality test, expressed as medians with upper and lower quartiles. Mann–Whitney and chi-square tests between groups showed statistical differences except for systolic blood pressure, preoperative PLT, PCO_2_, SaO_2_, Cr, and eGFR.

**Table 1:** Baseline characteristics of the study population.

Variable	Total (*n* = 1,017)	No transfusion (*n* = 595)	Transfusion (*n* = 422)	*Z*/*χ*^2^	*p*
Gender, *n* (%)				10.179	0.002
Male	421 (41)	271 (46)	150 (36)		
Female	596 (59)	324 (54)	272 (64)		
Age	49 (34, 64)	46 (33, 57)	53 (39, 69)	−6.221	<0.001
Wt	60 (52, 65)	60 (54, 68)	57 (50, 65)	−4.710	<0.001
SBP	110 (100, 122)	111 (104, 120)	110 (100, 125.75)	−1.820	0.069
DBP	68 (60, 77)	69 (61, 78)	68 (60, 75)	−2.645	0.008
SI	0.69 (0.61, 0.8)	0.68 (0.6, 0.75)	0.73 (0.63, 0.9)	−6.837	<0.001
HR	78 (70, 88)	75 (69.5, 82)	84 (73, 95.75)	−8.983	<0.00
ASAs, *n* (%)				208.626	<0.001
I–II	772 (76)	548 (92)	224 (53)		
III	171 (17)	39 (7)	132 (31)		
IV	74 (7)	8 (1)	66 (16)		
Temp	36.2 (36.2, 36.5)	36.2 (36.2, 36.2)	36.5 (36.3, 36.6)	−20.194	<0.001
LVEF	72 (66, 79)	71 (65, 78)	74 (67, 80)	−4.257	<0.001
FiO_2_	2 (2, 2)	2 (2, 2)	2 (2, 2)	−4.824	<0.001
EBL	50 (10, 200)	10 (10, 50)	200 (50, 800)	−19.183	<0.001
preHGB	125 (98, 143)	137 (124, 151)	94.5 (80, 112)	−21.203	<0.001
prePLT	191 (145, 241)	194 (149, 235)	188.5 (137, 257)	−0.007	0.995
PCO_2_	40 (36, 44)	40 (36, 44)	41 (37, 44)	−1.436	0.151
preINR	0.99 (0.95, 1.04)	0.98 (0.95, 1.02)	1.01 (0.96, 1.1)	−7.131	<0.001
preAPTT	28.7 (25.4, 33.6)	26.6 (24.3, 29.1)	33.2 (29.52, 37.1)	−16.217	<0.001
preFib	3.21 (2.62, 3.99)	3.28 (2.76, 3.88)	3.11 (2.34, 4.08)	−2.918	0.004
preTT	16.8 (16, 17.6)	16.9 (16.3, 17.7)	16.4 (15.6, 17.48)	−5.489	<0.001
preD-D	0.34 (0, 1.3)	0.17 (0, 0.47)	1.05 (0, 4.12)	−11.649	<0.001
TP	69.8 (63, 76)	73.4 (67.85, 77.6)	64.4 (55.5, 70.68)	−14.350	<0.001
ALT	20.7 (16.3, 28.3)	20.3 (16.6, 26.35)	21.8 (16.02, 34.68)	−3.102	0.002
TBIL	10.4 (7, 15.2)	11.1 (7.7, 16.1)	9.45 (6.12, 13.97)	−3.790	<0.001
Cr	56.2 (44.3, 74.9)	57.3 (44.4, 74.2)	54.9 (44.0, 75.3)	−0.028	0.978
eGFR	88 (72.9, 104.3)	89.1 (75.2, 104.3)	87.5 (66.8, 105.8)	−1.409	0.159
BG, *n* (%)				31.893	<0.001
A	287 (28)	148 (25)	139 (33)		
B	285 (28)	186 (31)	99 (23)		
O	321 (32)	167 (28)	154 (36)		
AB	124 (12)	94 (16)	30 (7)		
SG, *n* (%)				86.952	<0.001
I–II	104 (10)	77 (13)	27 (6)		
III	692 (68)	448 (75)	244 (58)		
IV	221 (22)	70 (12)	151 (36)		

ASAs, American Society of Anesthesiologists system; SI, Shock Index; LVEF, left ventricular ejection fraction; FiO₂, fraction of inspired oxygen; EBL, estimated blood loss (mL); BG, blood group; SG, surgical risk classification.

### 3.2 Lasso regression and cross-validation

**Figure 1:**
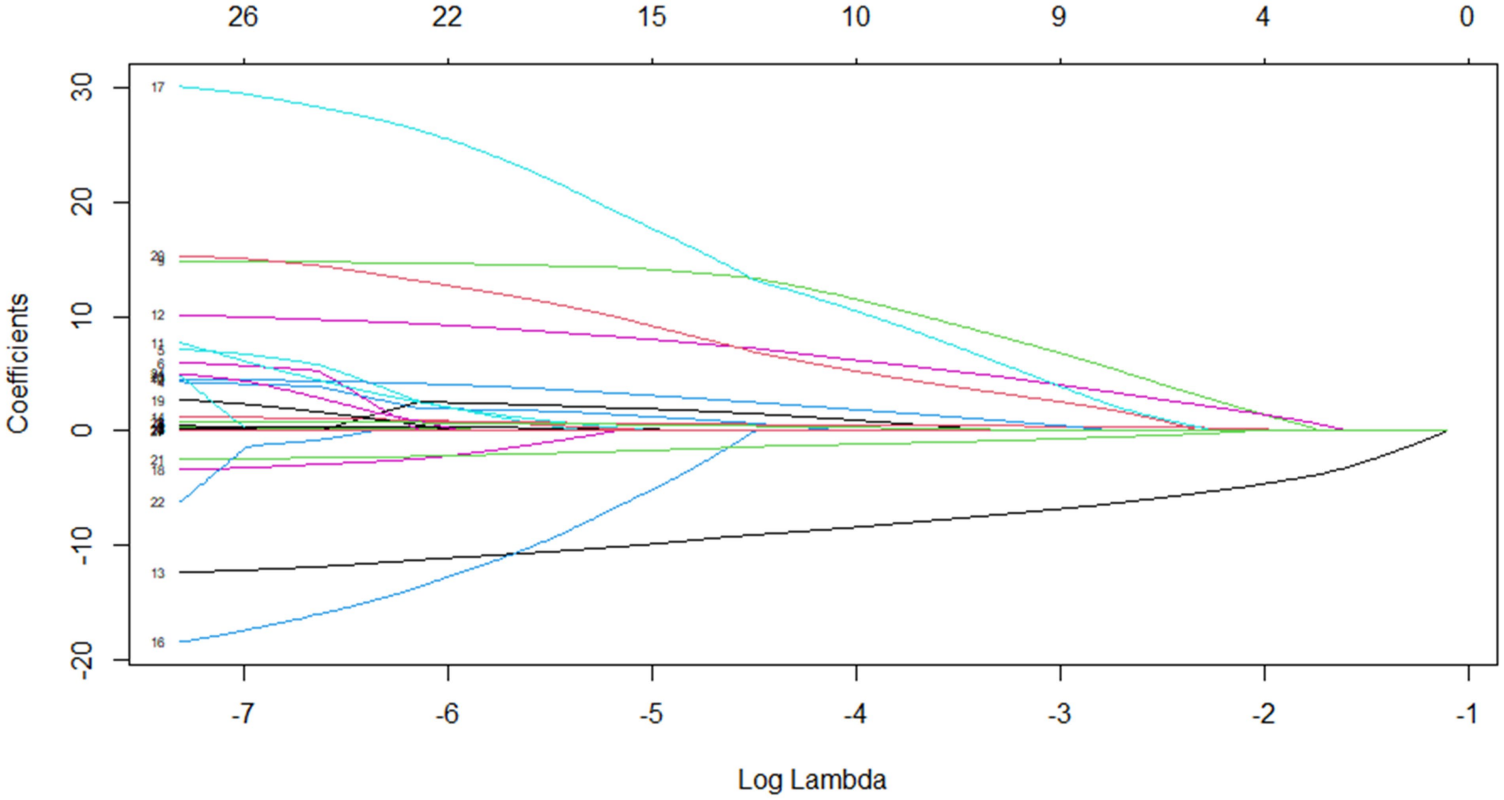
Lasso regression path diagram.

**Figure 2:**
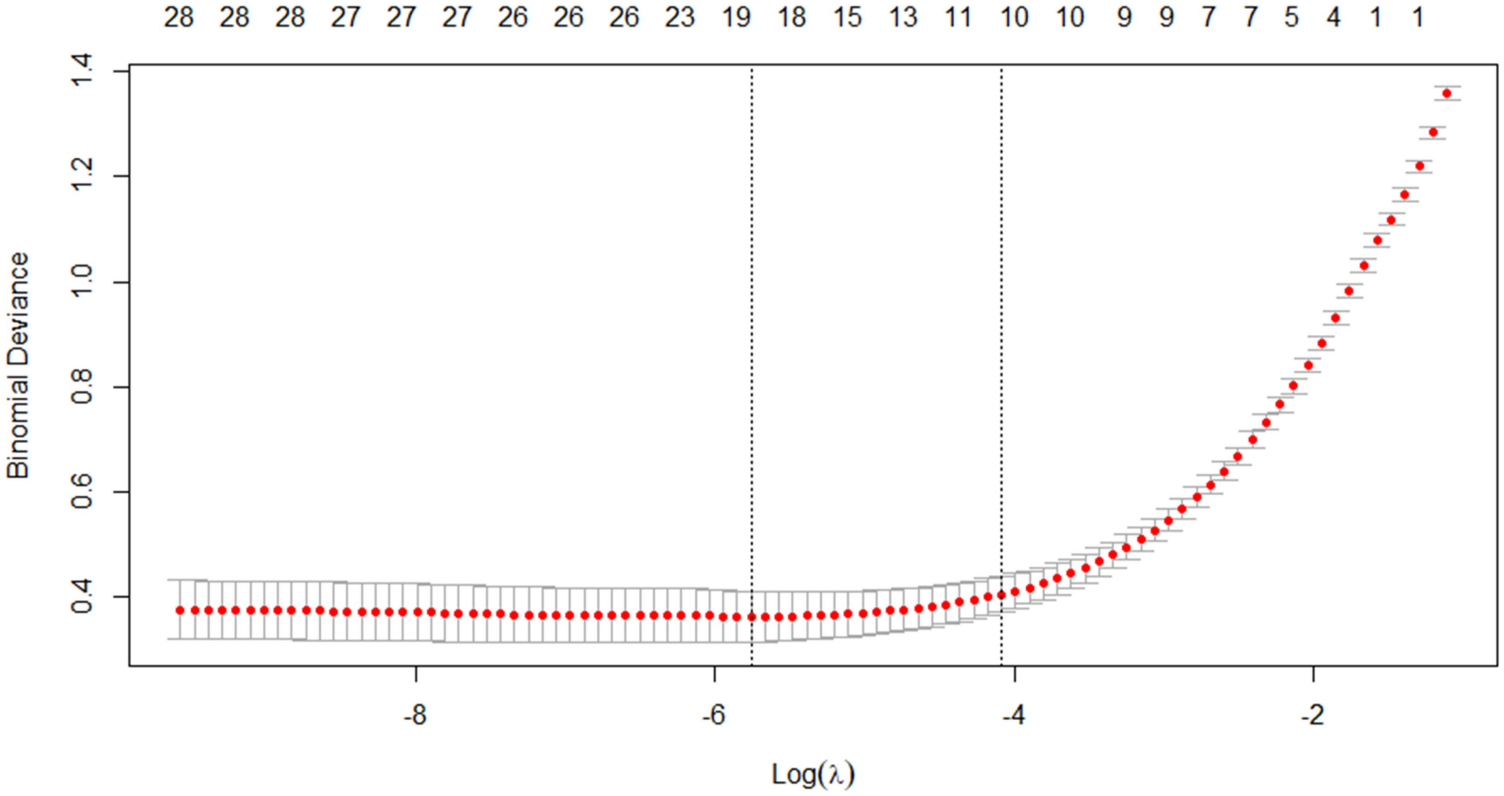
Lasso regression cross-validation results.

Through Lasso regression with cross-validation, the 11 potential risk factors that were most relevant to intraoperative blood transfusion of patients were screened out when Log (*λ*) took the value of 1SE, as shown in Table 2. The introduction of a logarithmically scaled penalty coefficient (*λ*) induces a sparsity-promoting mechanism: as the regularization strength increases, the magnitudes of the regression coefficients are progressively shrunk toward zero, with non-significant variables ultimately being excluded from the model when their coefficients reach exact zero. Lasso regression path diagram shown in Figure 1, Lasso regression with cross-validation shown in Figure 2. The final model retained 11 predictors by employing the optimal regularization parameter (*λ*) determined through one-standard-error rule, which selects the most parsimonious configuration within one standard error of the minimum binomial deviance observed during cross-validation.

**Table 2:** Lasso regression Log (*λ*) taking the value of 1SE.

Independent variable	Log (*λ*) = 1SE
(Intercept)	−0.267
Gender	/
Age	/
Wt	/
SBP	0.031
DBP	/
PI	/
HR	1.021
ASA	0.512
Temp	11.860
LVEF	1.948
FiO_2_	/
EBL	6.330
preHGB	−8.554
prePLT	/
PCO_2_	/
preINR	/
preAPTT	10.970
preFib	/
preTT	/
preDD	5.483
preTP	−1.215
preALT	/
preAST	/
preTBIL	/
preCr	/
preeGFR	/
BG	/
SurgicalGrade	0.311

### 3.3 Binary logistic regression modeling

**Figure 3:**
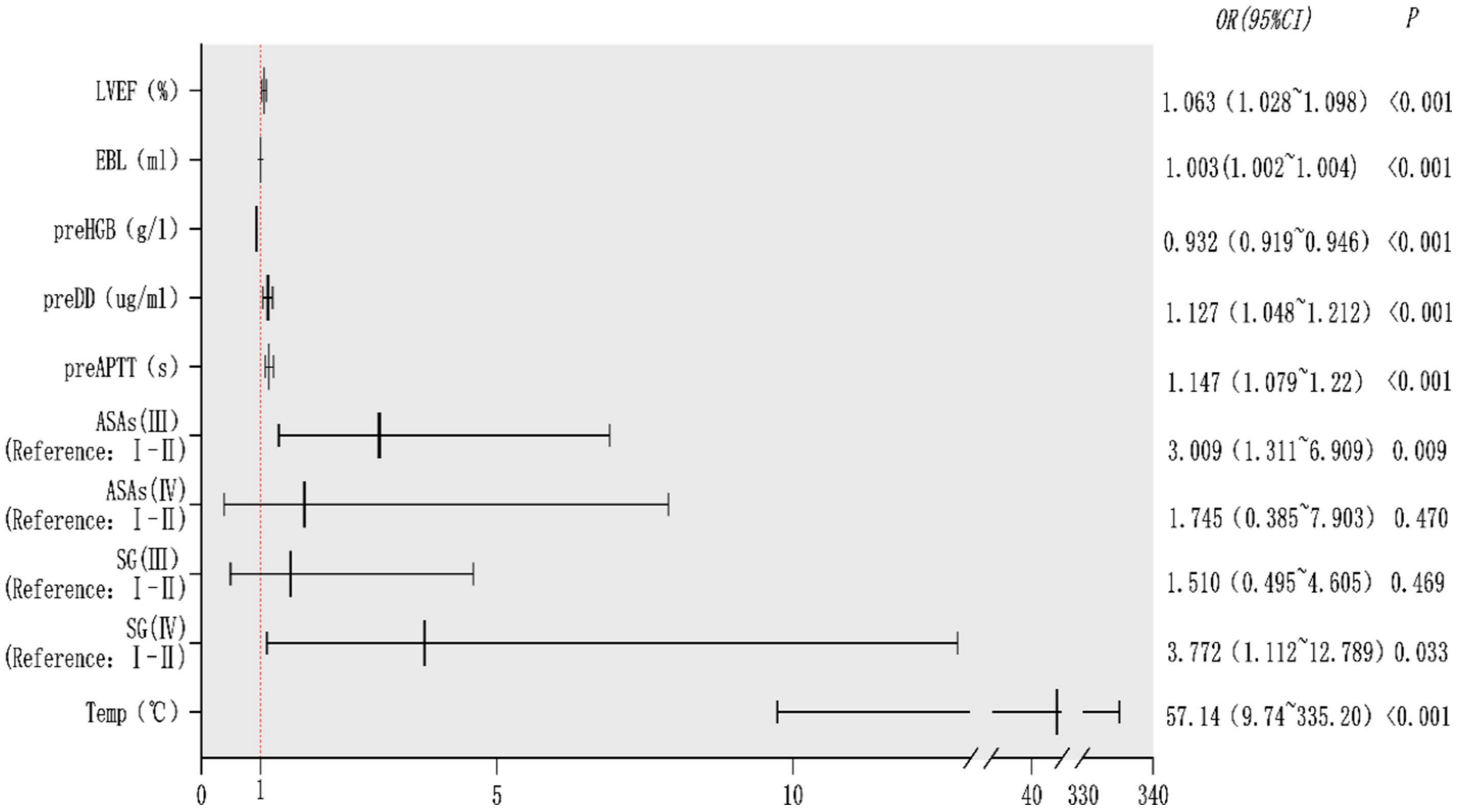
Forest plot of intraoperative blood transfusion risk.

**Figure 4:**
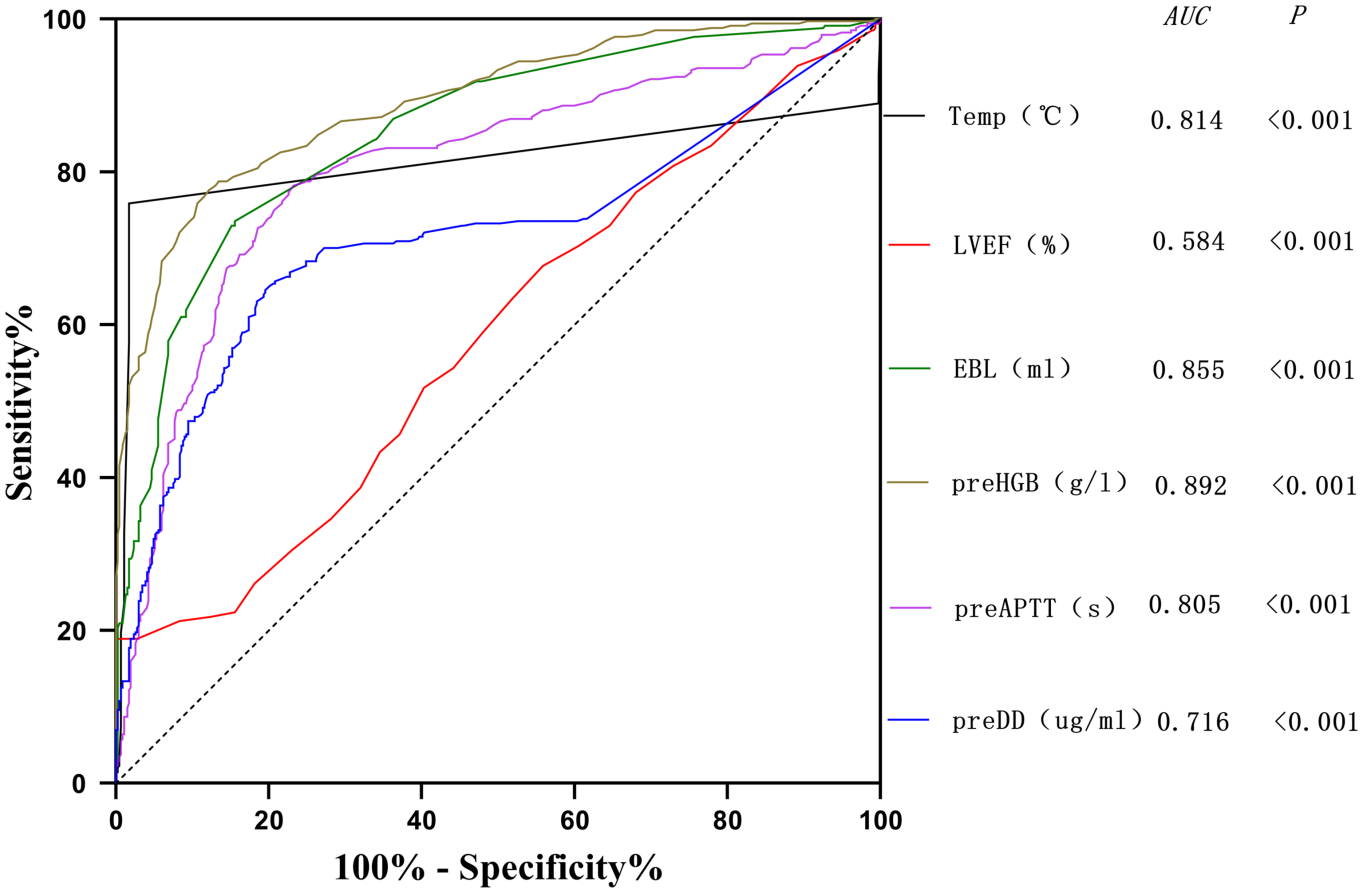
Multi-indicator independent diagnostic ROC curve.

**Figure 5:**
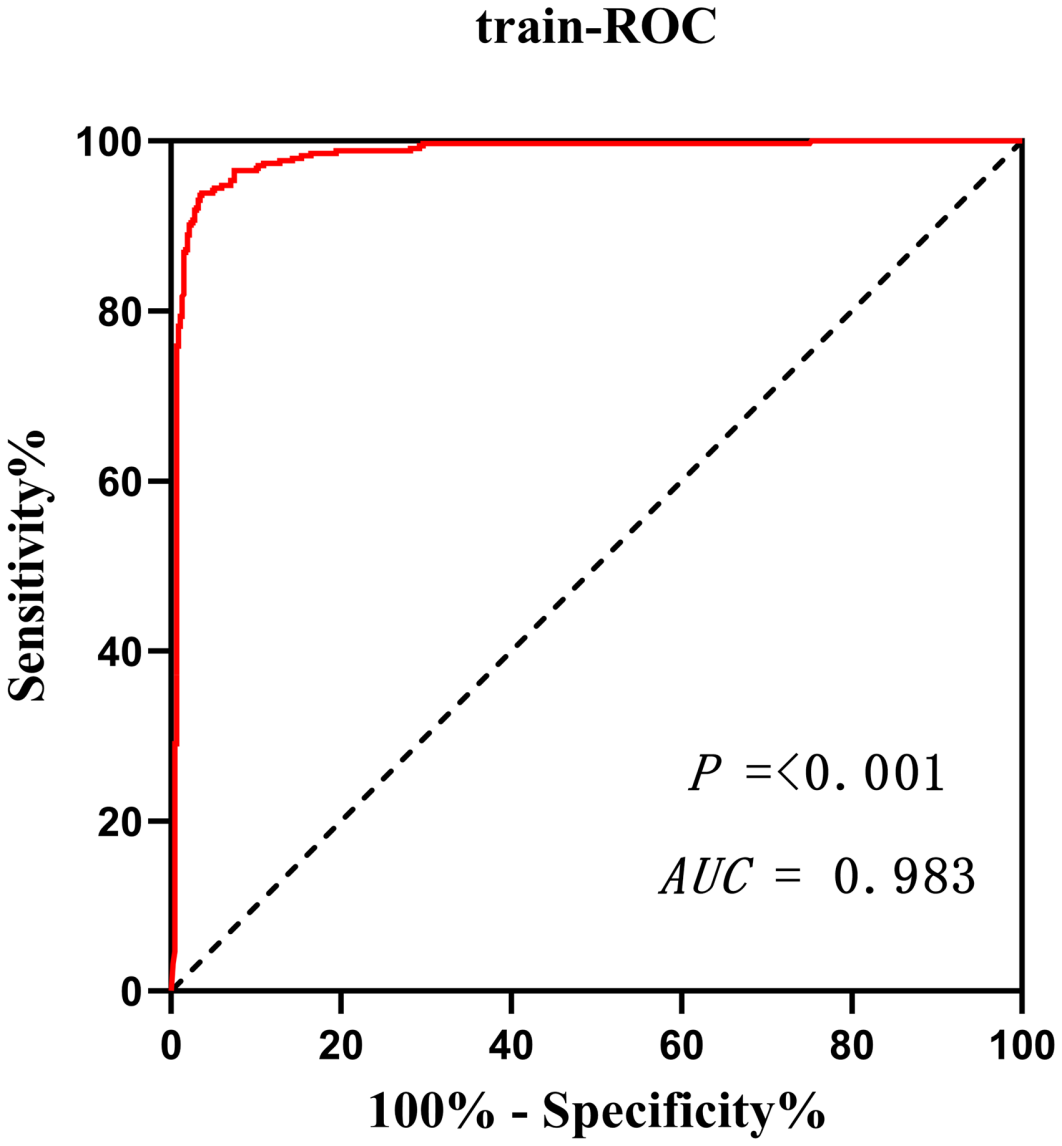
Multi-indicator combined diagnostic ROC curve.

The potential risk factors screened by Lasso regression analysis were introduced into binary logistic regression analysis to establish a regression model. Through the binary logistic regression analysis, the potential risk factors affecting patients’ intraoperative blood transfusion decisions were anesthesia classification (ASA III), surgical grade (SurgicalGrade IV), estimated intraoperative blood loss (EBL), preoperative hemoglobin concentration (preHGB), left ventricular ejection fraction (LVEF), intraoperative temperature (Temp), preoperative APTT (preAPTT), and preoperative D-D (preDD). A forest plot of intraoperative transfusion risk was plotted for visualization, as shown in Figure 3. Multi-indicator combined diagnostic ROC curves, as well as separate diagnostic ROC curves, were plotted for the model, and the area under the working curve (AUC) of the subjects was calculated separately. The Youden index was used to calculate the predictive probability threshold for the multi-indicator combined diagnostic curve, and the predictive probability threshold was 0.515, the sensitivity was 0.939, and the specificity was 0.964, as shown in Figures 4, 5.

### 3.4 Fitting a dynamic nomogram model

**Figure 6:**
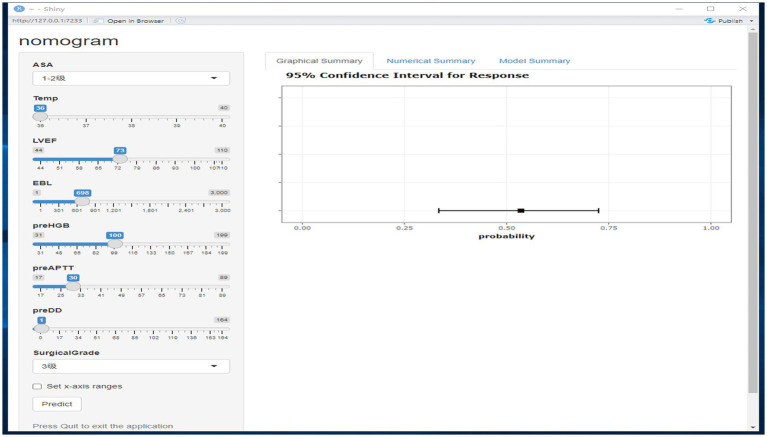
Intraoperative blood transfusion dynamic nomogram model.

The independent risk factors affecting patients’ intraoperative blood transfusion decisions derived from binary logistic regression were introduced into the RStudio statistical software; the random seed was set to 1, and the sample function was called to use 80% of the dataset as the training set and 20% as the test set. The training set data will be plotted in a dynamic nomogram model for intraoperative blood transfusion decision-making. We deployed the nomogram model on the web page through R software. By clicking on the website address on any terminal, the model can be opened. The web version of the model is shown in Figure 6. The categorical variables are selected through the drop-down menu, the continuous variables are selected by sliding the slider to select the value, and the probability of intraoperative transfusion of the patient, as well as the 95% CI, can be calculated by clicking on Predict, and when the probability is greater than the critical probability of 0.515, then the patient should be transfused to treat the intraoperative situation.

### 3.5 Model validation and evaluation

**Figure 7:**
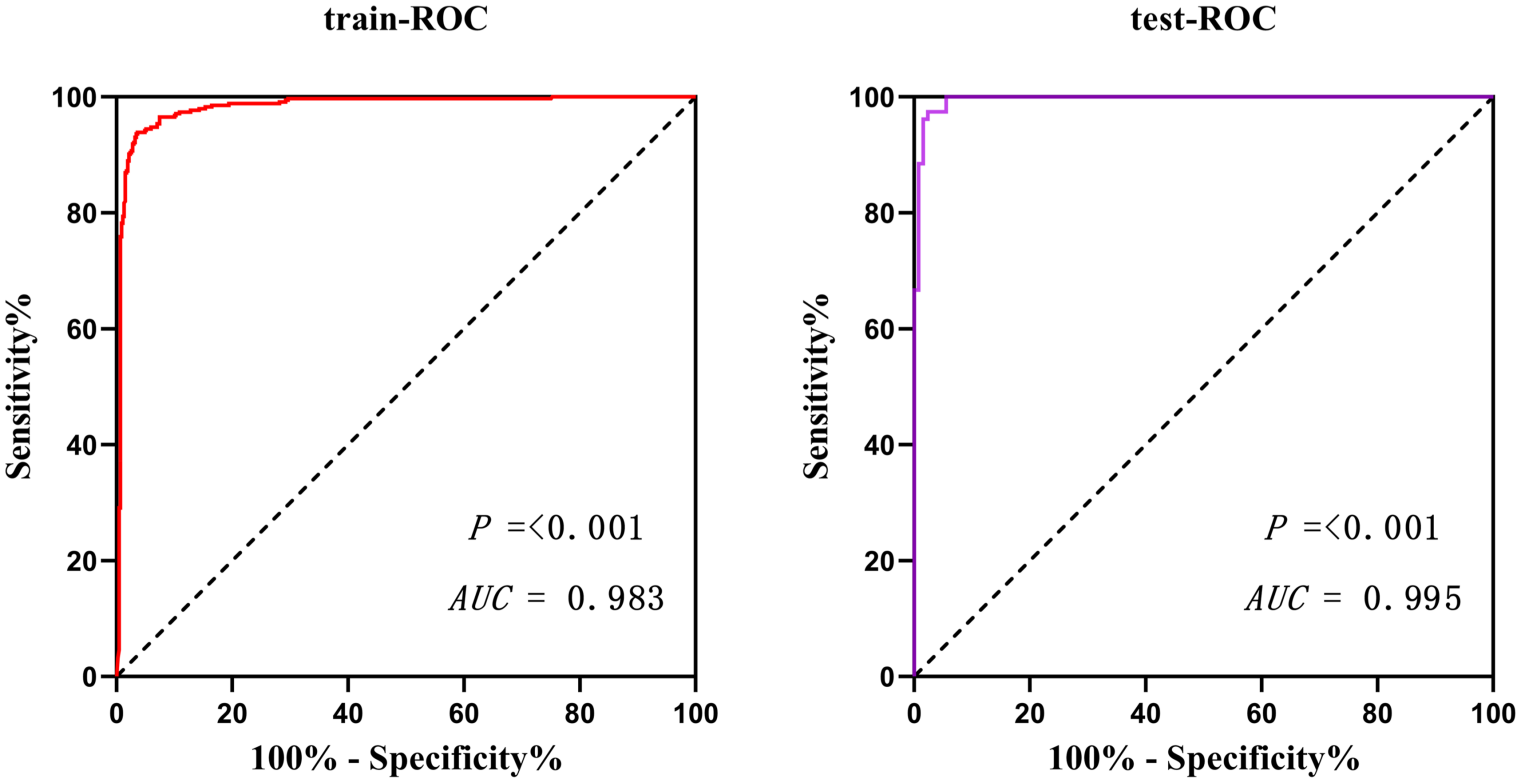
Model discrimination between training and test set.

**Figure 8:**
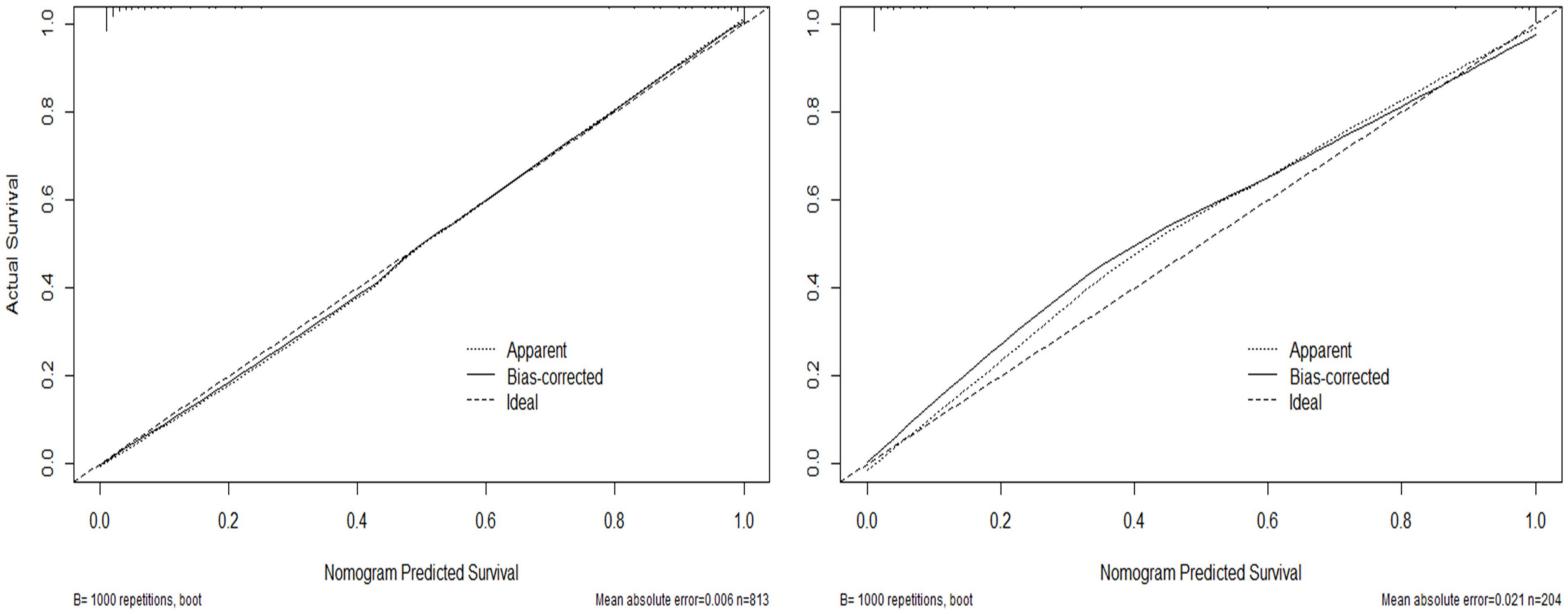
Training set and test set model calibration curve.

**Figure 9:**
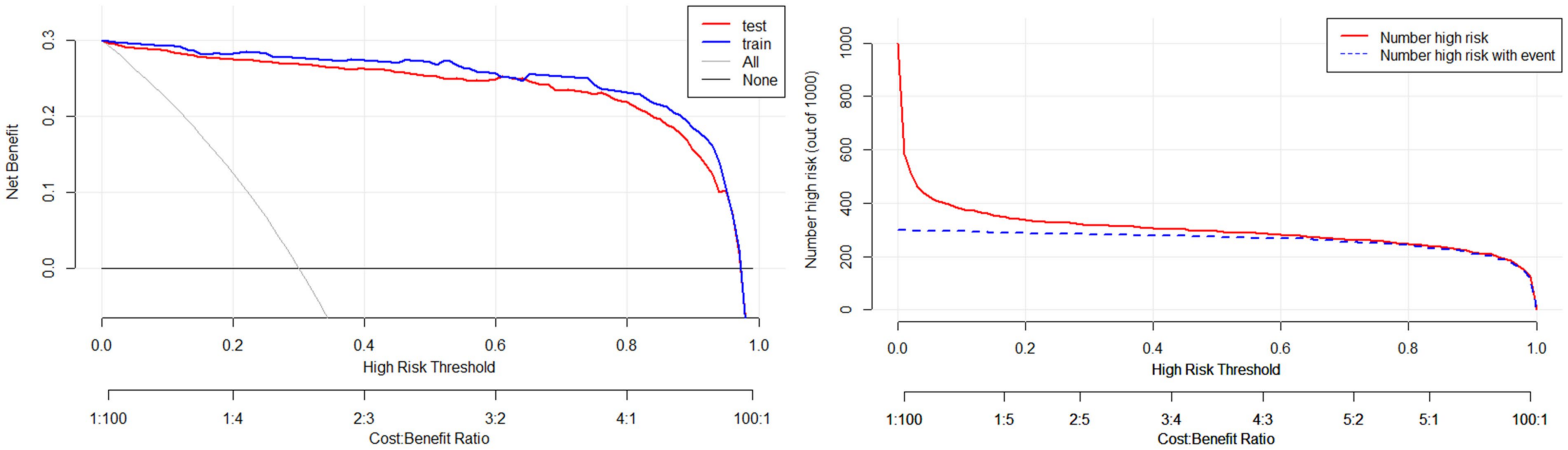
Clinical decision curve (left) and clinical impact curve (right) of the training set.

Validate the training set as well as the test set model, respectively. The Resource Selection package was loaded, and the Hosmer test was called to do the model fit goodness-of-fit test, *χ*^2^ = 111.85, *p* < 0.01. The AUC of ROC of the subjects was used to evaluate the model’s discriminative ability, and the AUC of the training set and the test set was 0.983 and 0.995, *p* < 0.05, respectively. The model discrimination is shown in Figure 7. Bootstrap was used to repeat the sampling 1,000 times to draw the calibration curve to evaluate the calibration of the model. The calibration curve showed that the predicted probability of the model and the observed probability were in good agreement; the calibration curve is shown in Figure 8. The clinical utility of the predictive model was rigorously evaluated through decision curve analysis (DCA) and clinical impact curves (CIC). As demonstrated in Figure 9, the decision curve revealed superior net benefit of the model-guided strategy compared to both the “treat-all” and “treat-none” extreme scenarios across a clinically relevant threshold probability range. Furthermore, the clinical impact curve quantified the projected clinical consequences, indicating that the model achieves a favorable balance between true-positive identifications and false-positive interventions at population scale.

## 4 Discussion

In this study, the 11 potential risk factors most related to intraoperative blood transfusion were first screened by Lasso regression and then analyzed by multifactorial binary logistic regression to find out the independent risk factors for intraoperative blood transfusion. Patients with anesthesia grades I and II had good anesthesia and surgical tolerance and a smooth anesthesia process. There is a certain danger in anesthesia for grade III patients, and the preparation before anesthesia should be sufficient, and effective measures should be taken for possible complications during anesthesia, and active prevention should be made. The anesthesia risk of grade IV patients was extremely high. Grade V and VI patients were not included in this study because of their extremely critical condition, poor tolerance of anesthesia, and the risk of death at any time. In the present study, anesthesia grade III was an independent risk factor for intraoperative blood transfusion, same as Patil et al. (13–15). However, Runge et al. (16, 17) showed that anesthesia grades III and IV were independent risk factors for intraoperative blood transfusion. This study only showed that anesthesia grade III was an independent risk factor for intraoperative blood transfusion. The reason for this may be that the data of this study came from a tertiary general hospital, the comprehensive strength is not enough, the critical patients have been transferred to the higher level of medical units, and anesthesia grade IV accounted for only 7% of the study cases. This study shows that surgical grading is also an independent risk factor for blood transfusion. 4-grade surgeries are defined as those that are high-risk and complicated in process, difficult, with increased difficulty of invasive intraoperative procedures and increased need for blood transfusion. Liu et al. (18), in his study of blood transfusion in major emergency abdominal surgery, also found that the type of surgery is a risk factor for perioperative transfusion. Intraoperative bleeding is unavoidable, and large intraoperative blood loss easily leads to a drop in blood pressure, decreased blood volume, increased heart rate compensation, and excessive blood loss will lead to excessive loss of red blood cells, platelets, coagulation factors, and other important components of the body, leading to hemodilution and coagulation disorders and entering a vicious cycle of blood loss and coagulation disorders. Intraoperative blood loss increases the need for intraoperative transfusion, and the study in this paper is similar to the existing studies (19–21) that intraoperative blood loss is an independent risk factor for intraoperative transfusion in patients. Patients with preoperative anemia exhibit lower hemoglobin levels and insufficient oxygen-carrying capacity of the blood, leading to tissue hypoxia, which makes patients less tolerant during surgery and exposes them to higher risks of cardiovascular risk, infection risk, and increased postoperative complications. Surgical blood loss directly leads to a further increase in blood volume and anemia, and severe anemia may lead to severe intraoperative hypoxia, increasing the risk of patient death. Preoperative anemia greatly increases the probability of intraoperative blood transfusion in patients and is an independent risk factor for intraoperative blood transfusion. Erben et al. (22), Attawettayanon et al. (23), and other researchers also concluded that preoperative anemia is an independent risk factor for intraoperative blood transfusion in patients. The left ventricular ejection fraction (LVEF) is an important index for assessing the pumping function of the heart, and its decline may be associated with cardiovascular disease, myocardial infarction, and other pathologies. Decreased LVEF leads to insufficient oxygen supply to vital organs throughout the body, and patients may exhibit increased heart rate and decreased blood pressure. The vital organs are hypoxic and have a decreased tolerance capacity, thus increasing the probability of intraoperative blood transfusion in patients. Some researchers have suggested a correlation between LVEF <35% and intraoperative blood transfusion in patients (24), which is similar to the findings of this paper. Intraoperative hypothermia is common in patients undergoing surgery, and medications, trauma, ambient temperature, type of anesthesia, and the extent and duration of the procedure can affect core temperature. Perioperative hypothermia has a greater impact on patient coagulation and increases the risk of intraoperative blood loss. Elevated perioperative body temperature accelerates metabolism and also increases the patient’s need for blood transfusion. Therefore, aggressive preoperative, intraoperative, and postoperative temperature management is needed to minimize the risk of perioperative hypothermia or hyperthermia. Simon Rauch (25, 26) and others concluded that intraoperative hypothermia increases the risk of intraoperative blood transfusion and is an independent risk factor for intraoperative blood transfusion. In this paper, we showed that elevated intraoperative body temperature increases the risk of intraoperative blood transfusion and that elevated body temperature increases the patient’s metabolic rate and oxygen consumption, leading to significant hypoxia and thus increasing the probability of transfusion. Guerra-Londono et al. (27) concluded that blood transfusion in patients undergoing hyperthermic intraperitoneal heat infusion chemotherapy (HIPEC) is associated with hyperthermia (≥39°C). In contrast to the perioperative transfusion pointer scale (POTTS), the transfusion trigger also increased with higher central body temperature and increased transfusion requirements. During surgery, coagulation indices play an important role in decision-making for intraoperative blood transfusion, and the present study showed that prolonged preoperative APTT and elevated preoperative D-dimer were independent risk factors for intraoperative blood transfusion. Kim et al. (28) study concluded that an INR greater than 1.2 was an independent risk factor for intraoperative blood transfusion. The important predictors of intraoperative blood transfusion in cesarean delivery were found in the model, including surgical method, surgical site, and coagulation-related indexes by Chen et al. (29). There are more factors influencing intraoperative blood transfusion, and previous studies have been seen. Arshi et al. (15) suggested that age, female sex, and low body weight are predictors of blood transfusion after hip fracture surgery in the elderly. Stoleriu et al. (30), in a retrospective cohort study in a high-volume thoracic surgical center, found that perioperative allogeneic transfusion in patients undergoing resection for primary lung cancer risk factors were female, platelet count, and RhD blood group. Pardessus et al. (31) concluded that increased intraoperative crystalloid fluid intake was a major predictor of intraoperative allogeneic transfusion in adolescents undergoing surgery for idiopathic scoliosis. Lee et al. (32) concluded that age greater than 85 years and type 2 diabetes mellitus were risk factors for intraoperative blood transfusion in total shoulder arthroplasty. Wagner et al. (33) also found smoking to be an independent risk factor for intraoperative blood transfusion. Walczak et al. also found creatinine to be a predictor of perioperative blood transfusion.

In this paper, we included the risk factors that previous researchers believed might affect intraoperative blood transfusion, and based on Lasso and multifactorial regression analysis, we integrated multiple predictive indicators and then fitted a dynamic nomogram with the help of R statistical software so that we could quickly and continuously calculate the probability of transfusion according to the patients’ ever-changing indicators during the operation to achieve individualized and precise guidance for blood transfusion. Our model can address the limitations of restrictive transfusion strategies by integrating dynamic personalized parameters. While restrictive strategies reduce unnecessary transfusions, they often ignore key factors such as cardiopulmonary function, metabolic rate, and intraoperative variables. Our nomogram model incorporates preoperative hemoglobin (preHGB), ASA, surgical classification, estimated blood loss (EBL), left ventricular ejection fraction (LVEF), and coagulation markers (preAPTT, preDD), which are capable of real-time adjustments based on patient physiology. For ASA type III patients, our model accounts for the reduction in physiologic reserve by weighting variables such as LVEF and intraoperative temperature (Temp). For example, ASA type III patients with elevated temperature (indicating a hypermetabolic state) or low LVEF (impaired cardiac output) may require blood transfusion when hemoglobin levels are above a restrictive threshold to ensure adequate oxygen delivery. In contrast, hemodynamically stable ASA III patients with minimal bleeding can safely avoid transfusion even when hemoglobin levels approach 70 g/L. This subtle approach reduces under-transfusion (risk of hypoxia) and over-transfusion (risk of complications). For patients with low hemoglobin (preHGB), the model dynamically adjusts recommendations by integrating EBL and coagulation status. A patient with a preHGB of 80 g/L may not need a transfusion if he or she has low bleeding and normal coagulation, whereas a patient with the same preHGB may need a transfusion if he or she has a significantly increased EBL and a prolonged preAPTT. The model may reduce unnecessary transfusions: in patients with adequate compensatory mechanisms, e.g., high LVEF and stable body temperature, even with a slightly lower preHGB, the model may suggest postponing transfusion and reducing transfusion-related risks. However, the data in this paper come from a single-center hospital, the modeling sample size is small and there may be sampling errors; the types of surgical diseases are small, and the proportion of level IV surgeries is low; in addition, the accuracy of the estimation of intraoperative blood loss depends on the doctor’s experience, and the blood loss in the anesthesia record is often lower than the actual amount of blood loss. In addition, the intraoperative temperature in this paper is the temperature at the time of blood transfusion, which may not reflect the fluctuation of the patient’s temperature throughout the operation. To improve the accuracy of the model, first, to address potential sampling bias and improve generalizability, we will integrate additional intraoperative variables in future iterations of the model, including dynamic vital sign trends (e.g., hemodynamic fluctuations), advanced coagulation profiles (e.g., thromboelastometry), oxygen balance metrics (e.g., mixed venous oximetry), autologous blood recovery, and anticoagulant regimens. Second, to minimize geographic and institutional bias, we are collaborating with hospitals in different regions (Asia, Europe, and North America) and in different healthcare settings (tertiary centers, community hospitals) to compile a multicenter dataset that includes heterogeneous surgical populations, rare procedures, and underrepresented populations. Finally, we are designing a rigorous external validation framework to assess the model’s performance in different transfusion practices (restrictive vs. liberal strategies), surgical specialties (e.g., trauma, oncology), and resource-limited settings. This multi-stage effort will not only validate the model’s discriminatory power and calibration, but will also ensure its adaptation to global clinical workflows. We are committed to reporting these advances in subsequent publications, with the ultimate goal of providing a universally applicable tool for precision transfusion medicine.

## Data Availability

The raw data supporting the conclusions of this article will be made available by the authors, without undue reservation.
