# Supplementary material for: Development and evaluation of a dynamic nomogram model for intraoperative blood transfusion decision-making
Source: Front Med (Lausanne). 2025 Jun 13;12:1566325. doi: 10.3389/fmed.2025.1566325 (PMC12202218; doi:10.3389/fmed.2025.1566325)
Supplement: Supplementary file 1 [file Data_Sheet_1.docx]

**Supplementary material**


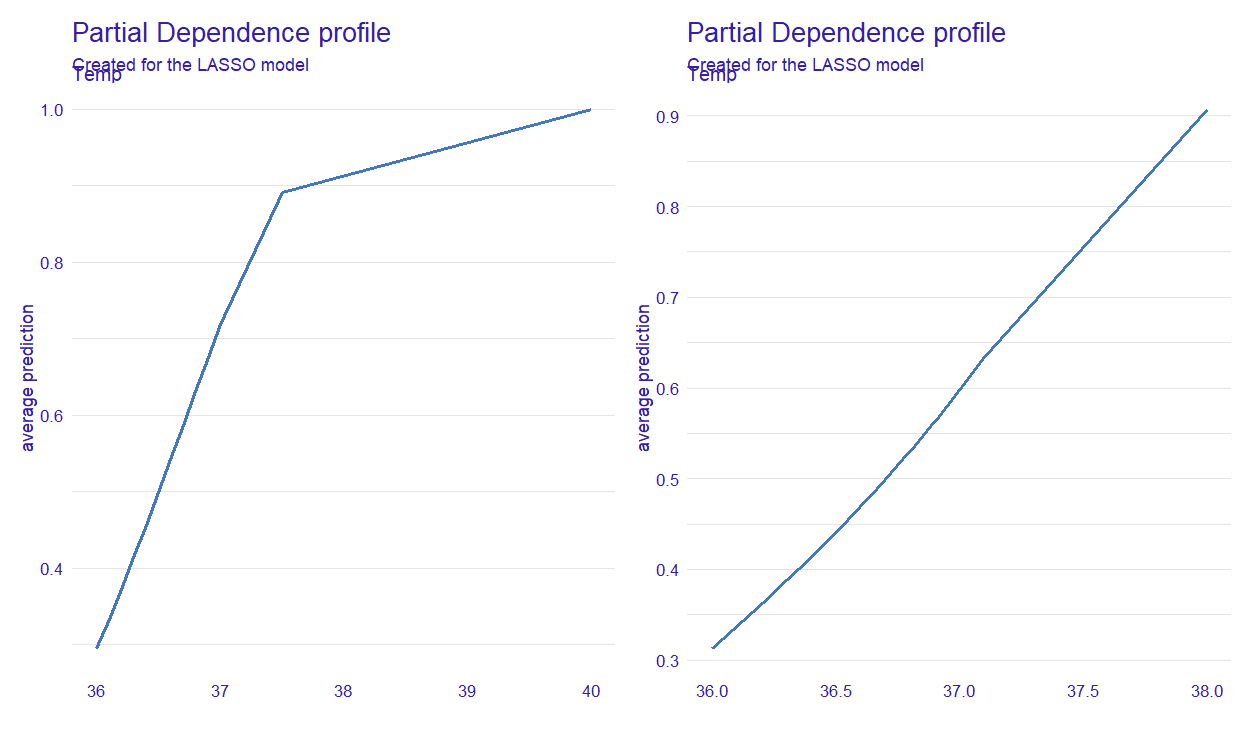


Suppl. Fig. 1: Temp-biased dependency graph between the training set and the test set

We created a nomogram of the added cases to explain the application of the model. See Suppl. Fig. 2. The scores corresponding to the patient’s individual risk factors, as well as the corresponding overall probability of intraoperative transfusion, can be clearly seen in Suppl. Fig. 2.


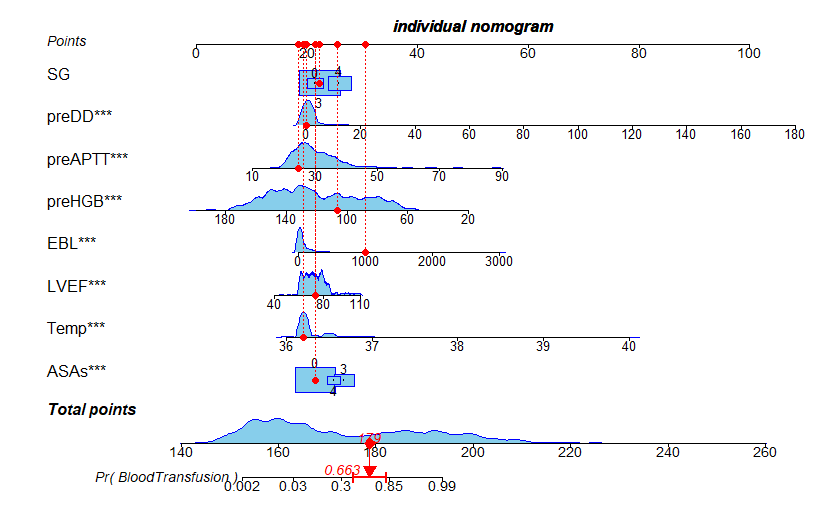


Suppl. Fig. 2: Individual nomogram


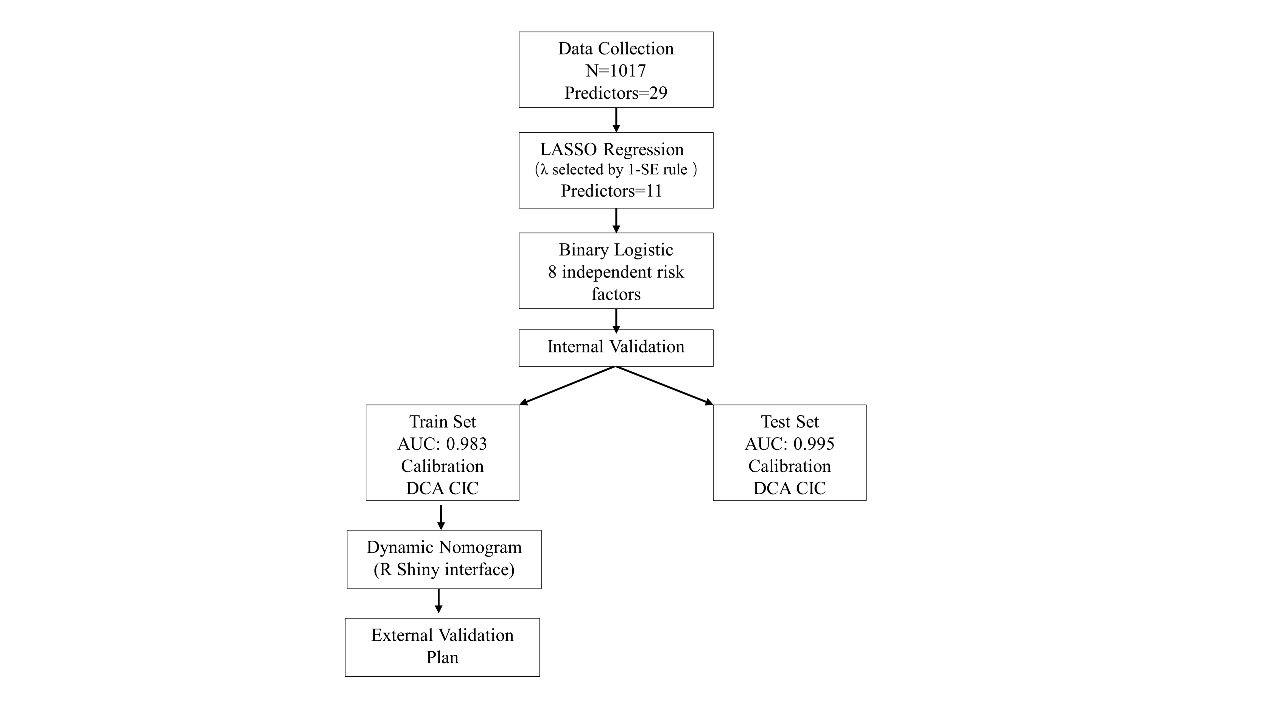


Suppl. Fig. 3: Statistical Flowchart
